# Spatiotemporal Control of Neuronal Remodeling by Cell Adhesion Molecules: Insights From *Drosophila*

**DOI:** 10.3389/fnins.2022.897706

**Published:** 2022-05-12

**Authors:** Hagar Meltzer, Oren Schuldiner

**Affiliations:** ^1^Department of Molecular Cell Biology, Weizmann Institute of Science, Rehovot, Israel; ^2^Department of Molecular Neuroscience, Weizmann Institute of Science, Rehovot, Israel

**Keywords:** pruning, cell adhesion molecules, *Drosophila*, neuronal remodeling, wiring and pruning, IgSF

## Abstract

Developmental neuronal remodeling is required for shaping the precise connectivity of the mature nervous system. Remodeling involves pruning of exuberant neural connections, often followed by regrowth of adult-specific ones, as a strategy to refine neural circuits. Errors in remodeling are associated with neurodevelopmental disorders such as schizophrenia and autism. Despite its fundamental nature, our understanding of the mechanisms governing neuronal remodeling is far from complete. Specifically, how precise spatiotemporal control of remodeling and rewiring is achieved is largely unknown. In recent years, cell adhesion molecules (CAMs), and other cell surface and secreted proteins of various families, have been implicated in processes of neurite pruning and wiring specificity during circuit reassembly. Here, we review some of the known as well as speculated roles of CAMs in these processes, highlighting recent advances in uncovering spatiotemporal aspects of regulation. Our focus is on the fruit fly *Drosophila*, which is emerging as a powerful model in the field, due to the extensive, well-characterized and stereotypic remodeling events occurring throughout its nervous system during metamorphosis, combined with the wide and constantly growing toolkit to identify CAM binding and resulting cellular interactions *in vivo*. We believe that its many advantages pose *Drosophila* as a leading candidate for future breakthroughs in the field of neuronal remodeling in general, and spatiotemporal control by CAMs specifically.

## Introduction

Following their initial establishment, developing neural circuits are further refined by a combination of degenerative and regenerative events. Collectively known as developmental neuronal remodeling, such processes are essential for shaping the connectivity of functional circuits, and represent a conserved strategy occurring throughout the animal kingdom and across the peripheral and central nervous systems. Remodeling varies in scale, from retraction of single synapses, up to degeneration of long stretches of axons or dendrites, often with remarkable spatiotemporal precision. Regressive steps are generally followed by progressive ones including stabilization and even reformation of new, adult-specific connections (Luo and O’Leary, 2005; Riccomagno and Kolodkin, 2015; Schuldiner and Yaron, 2015; Yaniv and Schuldiner, 2016). Defects in the normal progression of remodeling have been implicated in various neurodevelopmental and neuropsychiatric conditions, such as schizophrenia, autism spectrum disorder and Alzheimer’s disease (Cocchi et al., 2016; Hong et al., 2016; Sekar et al., 2016; Thomas et al., 2016). Despite constant progress, the molecular mechanisms underlying remodeling, and specifically its spatiotemporal control, remain poorly understood.

In recent years, it is becoming increasingly evident that neuronal remodeling is not solely governed by intrinsic genetic programs and cell-autonomous mechanisms (reviewed in Riccomagno and Kolodkin, 2015; Schuldiner and Yaron, 2015; Rumpf et al., 2019), but is also highly dependent on interactions with the environment – whether other neurons, non-neuronal cells or the extracellular matrix (Meltzer and Schuldiner, 2020). Moreover, recent studies have highlighted the importance of orchestrated circuit remodeling, in which different neuronal types in a given network simultaneously remodel in an interdependent manner (Mayseless et al., 2018; Lee and Doe, 2021). Due to their location on plasma membranes, cell adhesion molecules (CAMs) are prime candidates to mediate cell–cell interactions during coordinated circuit assembly and remodeling. Indeed, during initial steps of circuit formation, such as axon pathfinding and fasciculation, the role of CAMs, and other cell surface and secreted proteins (CSSPs), is relatively established (e.g., Dickson, 2002; Pollerberg et al., 2013). However, much less is known about the function of CAMs in regulating the spatiotemporal precision of developmental remodeling. Arguably, circuit reassembly during remodeling, occurring at late developmental stages, in larger neurons and for specific neuronal components, provides an excellent opportunity to deduce about similar mechanisms of initial circuit formation, which is less experimentally accessible at least in part due to its spatiotemporally “dense” nature.

Here, we explore recent advances in uncovering how CAMs and other CSSPs shape neuronal remodeling – including both neurite pruning and subsequent regrowth – in the fruit fly, *Drosophila melanogaster* (notably, for the sake of simplicity, the term “CAMs” is loosely used hereafter, as in some cases it refers to CSSPs of families generally known to be associated with adhesion, even when an adhesive role was not directly established). Of course, focusing on *Drosophila* does not underestimate the significant contributions of research in mammalian models, mostly to understanding the roles of CAMs in synapse retraction/stabilization (reviewed in Duncan et al., 2021). However, we believe that *Drosophila* holds major advantages that position it as an ideal model for substantial progress in the field. First, as a holometabolous insect, its entire nervous system is dramatically and stereotypically reorganized during metamorphosis. Indeed, many of its central and peripheral circuits undergo remodeling, and these are often well-characterized in terms of anatomy, development, and function (Yu and Schuldiner, 2014; Yaniv and Schuldiner, 2016). Second, and more importantly, *Drosophila* offers a particularly wide, and continuously expanding, arsenal of cutting-edge tools and techniques. Most pronounced is the ability to genetically access and perturb almost every neuronal type, but more recent advances in genomic tools, and in delineating protein interaction networks (“interactomes”; e.g., Ozkan et al., 2013), combined with the virtually complete EM-based connectome data of the fly brain (Scheffer et al., 2020), are now providing solid ground for delving into the mechanisms underlying neuronal remodeling and (re)wiring at up to subcellular resolution. Finally, relevant genes and pathways are largely conserved, and many important mammalian neuronal CSSPs have orthologs, or were even originally discovered, in *Drosophila*. Furthermore, neurodevelopmental processes, such as the molecular mechanisms of axon guidance and target selection, as well as neural organizational principles, such as the logical flow in the olfactory system, show striking similarity between flies and mammals (Komiyama and Luo, 2006; Reichert, 2009; Gonda et al., 2020; Li F. et al., 2020; Malin and Desplan, 2021). Thus, insights and principles obtained in *Drosophila* are likely to be relevant to similar processes in higher organisms.

## Transcription of Cell Adhesion Molecules Is Highly Dynamic During Development

Cell adhesion molecules that are required in specific locations at distinct time-windows could potentially have different or even deleterious effects if expressed in ectopic locations or developmental stages. Thus, precise CAM expression, in the right place and time, must be tightly regulated. Recent advances in high-throughput RNA-sequencing technologies provided the opportunity to map the transcriptional profiles of developing neurons (Alyagor et al., 2018; McLaughlin et al., 2021; Ozel et al., 2021; Xie et al., 2021; Janssens et al., 2022), thus revealing the temporally dynamic expression of CAMs and other CSSPs.

**FIGURE 1:**
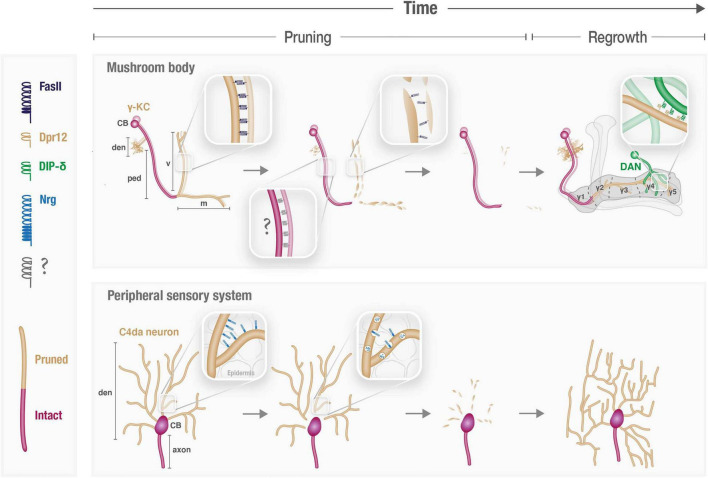
Cell adhesion molecules participate in spatiotemporal control of neuronal remodeling. Schematic illustration of the known and speculated roles of CAMs during pruning and regrowth of the γ-Kenyon cells (KCs) in the mushroom body (MB) and peripheral sensory Class 4 (C4) da neurons. CB, cell body; den, dendrites; ped, axon peduncle; v and m, vertical and medial axonal branches, respectively.

The *Drosophila* mushroom body (MB) is a well-characterized circuit in the fly brain that is comprised of three types of intrinsic neurons, known as Kenyon cells (KCs), which are sequentially born from the same neuroblasts. The first-born KCs – called γ-KCs – undergo stereotypic remodeling during metamorphosis, in which they prune their dendrites completely, and their bifurcated axons up to their branchpoint. Later during the pupal stage, γ-KCs regrow their dendrites, and their axons to form adult-specific projections (Lee et al., 1999; Yaniv and Schuldiner, 2016; Figure 1). γ-KCs were recently sequenced at unprecedented temporal resolution, including every 3 hours during early pupal development (Alyagor et al., 2018). This γ-KC transcriptional atlas revealed the extremely dynamic nature of gene expression in general, and CAMs/CSSPs specifically, along development. In fact, the transcriptional landscape of adult γ-KCs resembles the landscape of other adult neurons more than that of γ-KCs during pupal development. A follow-up study, which focused on the genetic program of γ-axon regrowth, highlighted the dynamic expression of Immunoglobulin Superfamily (IgSF) proteins (Bornstein et al., 2021). Moreover, IgSFs were enriched among genes whose expression changed upon inhibition of regrowth. Among IgSFs, the expression of proteins of the Defective in proboscis extension response (Dpr) family was especially striking, as 16 out of the 21 family members are expressed in γ-KCs in temporally dynamic patterns. Dpr12, for example, is downregulated at the onset of metamorphosis (prior to pruning) but is later gradually upregulated, in a timeframe suitable for γ axon regrowth. Indeed, while Dpr12 was found to be redundant for axon pruning, it is critical for the subsequent phase of γ-KC remodeling – in which axons regrow to occupy the full extent of the γ-lobe (Bornstein et al., 2021). Thus, this study demonstrates how temporally resolved transcriptional datasets can be translated to analyses of protein function (see also later). Interestingly, several other Dprs are upregulated in time points that precede γ-axon pruning (Alyagor et al., 2018), suggesting members of the Dpr family play yet undiscovered roles in the pruning process, and not only during axon regrowth.

Other studies focused on revealing the transcriptomes of developing neurons in the *Drosophila* olfactory sensory circuit. In this system, olfactory sensory neurons (OSNs) expressing the same odorant receptor converge onto one of ∼50 discrete glomeruli in the antennal lobe – a structure analogous to the mammalian olfactory bulb – where they synapse with a single class of projection neurons in each glomerulus (PNs, which correlate to mammalian mitral cells and relay sensory information to higher brain centers). It was previously shown that embryonic-born PNs participate in both the larval and adult olfactory circuits, in which they innervate different glomeruli. Developmental studies indicate that during metamorphosis, PNs undergo local pruning of their dendritic and axonal terminal branches, followed by re-extension of adult projections. In contrast, larval-born PNs, which constitute the majority of PN types, only participate in the adult circuitry and do not remodel (Jefferis et al., 2002; Marin et al., 2005). CAMs of different families were shown to play key roles in determining wiring specificity in antennal lobe, by confining and segregating PN dendritic fields within specific glomeruli, as well as dictating PN-ORN synaptic matching (Hong and Luo, 2014). Recently, single-cell RNA sequencing of PNs was performed at four developmental stages (early/mid/late pupae and adult; Xie et al., 2021). Among the genes that were differentially expressed in all stages, CSSPs and transcription factors were the two most over-represented groups of proteins. CSSPs included many molecules that were previously implicated in neural wiring, such as Dprs, Dscam and Fasciclins. Interestingly, in the early pupal stage, PNs formed two distinct clusters, with the smaller cluster representing embryonically born PNs. Thus, the fact that these neurons undergo remodeling indeed reflects in significant transcriptomic changes, but how this correlates with CSSP expression is yet to be analyzed. Another recent study, which profiled the single-cell transcriptome of developing OSNs (McLaughlin et al., 2021), also revealed over-representation of CSSPs. Comparison of the PN/OSN datasets highlighted CSSPs that are broadly expressed in both, while others that are enriched in either OSNs or PNs. Uncovering PN/OSN ligand/receptor candidates should promote understanding not only of how their precise matching is achieved, but also of how OSNs facilitate refinement of PN dendrites following their glomeruli occupation, as was recently demonstrated by time-lapse imaging (Li et al., 2021).

Taken together, genomic and genetic studies in developing fly neurons imply that the full spectrum of functions played by CAMs/CSSPs during neural circuit pruning and (re)wiring are just beginning to be unraveled.

## The Membranal Availability of Cell Adhesion Molecules Is Spatiotemporally Regulated During Remodeling

**FIGURE 2:**
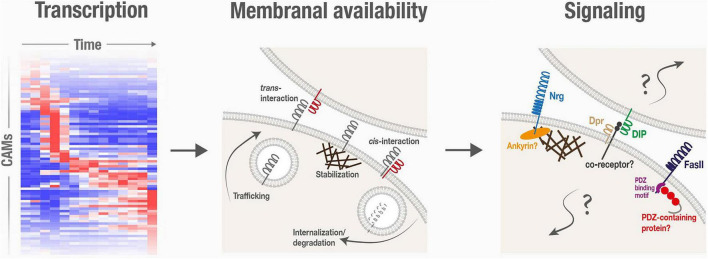
Cell adhesion molecules are spatiotemporally regulated, and are associated with poorly characterized signaling mechanisms, during neuronal remodeling.

Following transcription, the abundance and binding availability of CAMs on plasma membranes can be further regulated *via* cellular processes that affect delivery to the membrane (such as trafficking and exocytosis), stabilization within the membrane (such as interactions with the cytoskeleton) and, finally, removal from the membrane (such as endocytosis and degradation; Figure 2). Here we will describe some regulated alterations in membranal CAM expression that were shown to underlie spatiotemporal specificity of neurite pruning and circuit reformation.

Reducing the membranal abundance of CAMs is a key step in the remodeling of *Drosophila* sensory dendritic arborization (da) neurons, which extend highly branched dendrites along the body wall. Proper dendritic coverage during the initial elaboration of da dendrites requires self-avoidance and tiling mechanisms which are both mediated by CAMs (including Dscam and integrins; Matthews et al., 2007; Han et al., 2012; Kim et al., 2012). During metamorphosis, da neurons of two classes (I and IV) prune their larval dendritic arbors by local fragmentation, while their axons remain intact, and later regrow adult-specific dendritic arbors (Yu and Schuldiner, 2014; Figure 1). Downregulation of Neuroglian (Nrg), the sole homolog of L1-type CAMs in *Drosophila*, was found to be required for dendrite pruning of class IV da neurons (Zhang et al., 2014; Figure 1). Nrg downregulation occurs *via* endocytosis, as evident by its redistribution from the plasma membrane to endosomal compartments at the onset of pruning. Accordingly, overexpression of Nrg within da neurons is sufficient to inhibit their pruning, while its loss leads to precocious pruning (i.e., at an earlier time point). This indicates that the temporal specificity of dendrite pruning is dictated, at least in part, by precise timing of Nrg internalization. Interestingly, while Nrg is expressed, and internalized to endosomes, in both axons and dendrites, its loss selectively affects dendrites, and does not “force” ectopic pruning of axons. Therefore, the mechanism underlying compartment-specific pruning, and whether and how it relates to Nrg, remains unclear. Perhaps Nrg downregulation renders da neurons more susceptible to pruning by reducing their adhesion – most likely to the epidermis – and another, Nrg-independent mechanism protects axons from a similar fate. Since its identification, many additional regulators of Nrg endocytosis-mediated pruning have been uncovered, including members of the secretory pathway, protein trafficking, and endo-lysosomal degradation (Wang et al., 2017, 2018; Zong et al., 2018; Kramer et al., 2019; Rui et al., 2020). However, while this machinery must be tightly regulated in time to ensure stereotypic pruning of da dendrites, how this is achieved is unclear. Notably, the binding partner of Nrg in this context, and the potential cellular interactions it mediates, remain to be identified. It is thus possible that spatiotemporal cues for pruning are contributed by the interacting cells, such as epidermal cells, which are known to engulf pruned debris, or glia, shown to be tightly associated with da dendrites near their proximal severing sites (Han et al., 2011). Interestingly, Nrg-mediated interactions were also shown to be required for synaptic stability in the developing fly neuromuscular junction (NMJ), as Nrg loss results in increased synapse pruning (Enneking et al., 2013). Furthermore, Nrg was implicated in the remodeling of the *Drosophila* Giant Fiber (GF) circuitry, which exhibits pruning of extraneous axonal branches during the pupal stage (Borgen et al., 2017). In this system, Nrg was shown to be retrogradely transported from GF terminals in an Amyloid Precursor Protein-like (APPL)-dependent manner (Kudumala et al., 2017; Penserga et al., 2019). APPL mutants exhibit pruning defects of GF transient branches, thus implying a potential role for Nrg in GF pruning, although this was not directly tested. Interestingly, mammalian L1-type CAMs, including NrCAM and CHL1, were implicated in adolescent spine pruning in mouse genetic models. However, unlike with *Drosophila* Nrg, NrCAM/CHL1 absence actually results in increased spine density (i.e., decreased pruning), as their interactions induce intracellular signals that eventually lead to spine collapse (Demyanenko et al., 2014; Mohan et al., 2019a,b). The underlying cause of these seemingly opposite outcomes, perhaps stemming from differences in the balance between adhesive and signaling functions, is yet to be resolved.

Downregulation of membranal CAM levels is also crucial during MB remodeling, in which γ axons must be defasciculated at the onset of metamorphosis to prune (Bornstein et al., 2015; Figure 1). This destabilization is achieved *via* c-Jun N-terminal Kinase (JNK)-mediated reduction in the membranal levels of the IgSF CAM Fascilin II (FasII), the ortholog of the mammalian neural cell adhesion molecule (NCAM). While trafficking was ruled out as the major regulator of FasII membranal levels, whether FasII downregulation also occurs *via* endocytosis, or by an alternative destabilizing mechanism, is unknown. Mutating JNK, or overexpressing FasII, is sufficient to inhibit pruning of γ axons, but not dendrites. Moreover, overexpressing other CAMs has a similar effect, suggesting that increased axo-axonal adhesion, in general, prevents normal progression of pruning. Manipulations of JNK or FasII are the first case of selective regulation of axon vs. dendrite pruning. Interestingly, endogenous FasII is indeed only expressed in γ axons and excluded form dendrites and cell bodies, which could, in theory, account for the observed phenotype of JNK mutants. However, even strong transgenic FasII overexpression, which was also localized to dendrites, did not inhibit dendrite pruning (Bornstein et al., 2015). Thus, the differential subcellular distribution of endogenous FasII within γ-KCs cannot alone account for the axon-specific pruning defect, and the full mechanism underlying its different effects on dendrites and axons remains undetermined. One option is that it stems from anatomical constraints, since γ dendrites are not tightly fasciculated as the axons. However, one cannot rule out the contribution of additional factors, such as potential involvement of other cell populations (neurons or glia) that occupy the axonal but not dendritic area, or vice versa. Notably, the fact that γ-axon pruning accurately stops at the axonal branchpoint and does not extend into the axonal peduncle (Figure 1) also remains unexplained. The involvement of neighboring cells, and/or another CAM type that maintains its membranal expression in the peduncle, are interesting directions for future investigation.

Another mechanism for plasma-membrane stabilization could be *via trans*-interactions with neighboring cells. The MB circuitry includes, in addition to KCs, input neurons (mostly PNs), output neurons (MBONs), and modulatory neurons that are mostly dopaminergic (DANs). MBONs and DANs innervate the KC lobes in a compartmentalized fashion thus dividing the MB lobes to discrete and functionally relevant sub-axonal zones (Tanaka et al., 2008; Aso et al., 2014; Figure 1). Finer examination of the Dpr12 regrowth phenotype (see previous section) revealed a specific lack of the γ4/5 zones. Dprs form an elaborate network of interactions – presumed to be adhesive in nature – with Dpr interacting proteins (DIPs; Carrillo et al., 2015; Cosmanescu et al., 2018). Indeed, Dpr12 was found to interact with DIP-δ, expressed in a sub-population of DANs (Bornstein et al., 2021), to mediate γ4/5 zone formation. GFP-fusion proteins indicate that both DIP-δ and Dpr12 are localized to the γ4/5 zones. Remarkably, misexpressing DIP-δ in DANs that target the γ3 zone leads to ectopic localization of Dpr12 in the γ3 zone within γ-KCs. Conversely, loss of DIP-δ resulted in diffuse Dpr12 mislocalization (Bornstein et al., 2021). This suggests that the subcellular membranal localization of Dpr12 along the γ-KC axon is instructed and/or stabilized by its transneuronal interactions with DIP-δ in neighboring DANs. Similar mechanisms for differential subcellular distribution along the membrane might also be employed by other CAMs and in other neurodevelopmental contexts.

Finally, once on the plasma membrane, binding availability is another potential layer for regulation. Interestingly, the growing body of transcriptomics and proteomics datasets of developing neurons highlight cases in which known CAMs and their interacting proteins are expressed within the same neurons. For example, in the γ-KCs, cognate Dpr/DIP pairs are expressed in overlapping temporal patterns (Bornstein et al., 2021), suggesting that they co-exist on the same membrane. Co-expression, and potentially consequent binding in *cis*, might inhibit *trans* interactions with adjacent cells, a phenomenon known as *cis*-inhibition. Alternatively, *cis* binding can induce an intracellular signaling response, i.e., *cis*-activation. *Cis*-interactions were reported for several CSSPs, including Notch and its receptors, Ephrins/Eph receptors and Semaphorins/Plexins (del Alamo et al., 2011; Nandagopal et al., 2019; Rozbesky et al., 2020; Cecchini and Cornelison, 2021), and are important for developmental processes such as tissue patterning. Whether *cis*-interactions occur and play a role in neuronal remodeling is currently unknown and warrants further investigations.

## Signaling Pathways Associated With Cell Adhesion Molecules During Remodeling Are Incompletely Understood

A major unresolved question in the context of CAMs in neuronal remodeling is how does signaling fit into the picture? Beyond their roles in forming and stabilizing cell–cell adhesive structures, CAMs often propagate signal transduction, regulating crucial cellular responses such as cytoskeletal dynamics, cell polarity, and transcription activation (e.g., Cavallaro and Dejana, 2011). Various findings strongly imply that CAM-triggered signaling events are also central to neuronal remodeling in *Drosophila*, but their precise nature is mostly obscure (Figure 2).

Fascilin II downregulation was shown to be required for pruning of γ-KCs, but the mechanism by which JNK negatively regulates its membranal stability/residence is unclear. Interestingly, the c-terminal PDZ binding sequence of FasII – known to mediate interactions with cytoplasmic PDZ-containing scaffold proteins – was found to be crucial for the JNK-FasII regulation (Bornstein et al., 2015). While it was shown that JNK is unlikely to directly phosphorylate FasII, it is possible that it phosphorylates the PDZ-containing protein, but its identity remains to be revealed. NCAM, the mammalian ortholog of FasII, was shown to be important for pruning of excess perisomatic synapses during postnatal development of the prefrontal cortex. In this case, the suggested mechanism involves a complex interplay with Ephrins/Eph receptors and signaling by Rho-associated protein kinase (Brennaman et al., 2013; Sullivan et al., 2016). It remains to be determined if similar molecular players in *Drosophila* participate in FasII-mediated signaling during MB axon pruning.

Cell adhesion molecule-associated signaling seems to also be important in later steps of MB remodeling, during axon regrowth and circuit reformation. If Dpr12/DIP-δ interactions are adhesive in nature, why do axons stop in their absence? Furthermore, in replacement experiments, while the DIP-α-Dpr6/10 interaction was sufficient to compensate for the absence of Dpr12-DIP-δ, replacing their interaction by the adhesive interactions of FasII was not (Bornstein et al., 2021). This suggests that matching pairs of the Dpr/DIP network, regardless of their specific identity, exert their function *via* signaling mechanisms that are beyond mere adhesion. Since Dpr/DIPs are either GPI-anchored or contain small intracellular domains (Cheng et al., 2019a), it is likely that co-receptors are involved in mediating downstream signaling, but the identity of these, at the moment, is a complete mystery. Elucidating the mechanisms of Dpr/DIP interactions can potentially also shed light on interactions mediated by their mammalian orthologs – the five members of the IgLON family (Cheng et al., 2019b), which are also implicated in neurodevelopment, and are associated with neuropsychiatric disorders (Karis et al., 2018; Fearnley et al., 2021).

Similarly, within the developing fly NMJ, loss of Nrg results in increased synapse retraction that cannot be compensated by overexpression of FasII (which has known roles in synapse stabilization; Packard et al., 2003), implying specific Nrg-mediated signaling. In this case, the Ankyrin-binding domain of Nrg is crucial for its function in synaptic stability, suggesting a spectrin/cytoskeleton-related mechanism (Enneking et al., 2013; Weber et al., 2019). Notably, recruitment of Ankyrins to the cytoplasmic domains of L1-type CAMs as a mechanism to stabilize synapses is conserved in mammals (Duncan et al., 2021). A similar mechanism might also be associated with the function of Nrg in pruning of da dendrites. In general, while disassembly of the cytoskeleton is well-established as an early step of pruning in both invertebrate and vertebrate neurons (Watts et al., 2003; Brill et al., 2016; Rumpf et al., 2019), the significance of CAM-cytoskeleton associations in this context are yet to be fully elucidated.

Modified assays for *in vivo* proximity-labeling were recently applied in *Drosophila* pupae for cell-surface proteomic profiling of developing PNs (Li J. et al., 2020). Similar assays could be employed in developing flies to reveal novel binding partners of specific proteins, by directly fusing the biotinylating enzyme to the endogenous protein of interest. Such assays, combined with the availability of multiple binary systems to simultaneously perturb and/or visualize distinct cell populations, should facilitate future identification and functional analysis of co-receptors and downstream effectors of signaling pathways associated with CAMs during remodeling.

## Discussion

Despite the fundamental significance of neuronal remodeling for the proper formation of mature neural circuits, our understanding of the mechanisms that regulate it is limited. Developments in the *Drosophila* toolkit facilitate gradual unraveling of the roles played by CAMs and other CSSPs during distinct remodeling processes, and highlight their potential contributions to timely execution, spatial precision and wiring specificity. Naturally, many open questions remain to be resolved before we can reach a comprehensive understanding of the various functions of CAMs during developmental remodeling.

A fascinating aspect in the field, which is only beginning to be unraveled, is the concurrent remodeling of different neuronal types within the same circuit. While CAMs are excellent candidates to coordinate such processes, their functions in this context are largely unknown. In the *Drosophila* MB, γ-KCs and the GABAergic anterior paired lateral (APL) neuron were shown to undergo developmental remodeling in the same timeframe. Moreover, cell-autonomous inhibition of γ-KC pruning disrupts APL pruning. This coordination relies on γ-KC activity, and Calcium/Calmodulin signaling within the APL neuron. Interestingly, artificially increasing γ-KC-to-APL adhesion by ectopically expressing FasII is sufficient to inhibit pruning of both neuronal types (Mayseless et al., 2018). Whether and how CAMs provide the spatiotemporal cues triggering orchestrated remodeling of neuronal circuits, and the regulatory interplay between CAM expression and neuronal activity in this specific context, are yet to be resolved. *Drosophila* is an ideal model to address such issues, due to its well-characterized circuits and the genetic handle to almost all cell types.

Another aspect that may revolutionize our understanding of neural network assembly is deciphering “adhesion codes” that underlie synaptic (re)wiring of complex and stereotypic circuits. In the MB, Dpr12 and DIP-δ mediate formation of the γ4/5 axonal zones during γ-KC regrowth (Bornstein et al., 2021), but they are just one pair out the many “Dpr-ome” members that are dynamically expressed in developing γ-KCs (Alyagor et al., 2018; Bornstein et al., 2021), while many DIPs are differentially expressed in DANs and MBONs (Croset et al., 2018; Aso et al., 2019). Thus, it is tempting to speculate that other Dpr/DIP combinations instruct the formation the remaining MB axonal zones, by encoding the match between DANs, MBONs, and KCs. Dpr/DIPs were demonstrated to mediate synaptic specificity in targeting of motoneurons to muscle fibers in the developing NMJ, for specific layer targeting in the visual system, and for positioning of OSNs to specific glomeruli in the olfactory system (e.g., Barish et al., 2018; Xu et al., 2018; Ashley et al., 2019; Menon et al., 2019). It therefore seems that similar molecular principles are employed for targeting of neurites to specific cell types/layers/structures during circuit assembly, and for specifying sub-axonal compartmentalization during circuit reassembly. Thus, studying the signaling mechanisms of Dpr/DIPs during MB circuit reassembly – occurring late in development in a genetically and visually accessible environment – provides an opportunity to also understand their function during initial circuit assembly in other neuronal systems. Moreover, Due to extensive biochemical and structural work (Cosmanescu et al., 2018; Sergeeva et al., 2020), the Dpr/DIP families also hold the promise to dissect how affinity variations between binding partners translate into their function during distinct steps of remodeling. Redundancy seems to be a complicating factor, as many of the Dpr/DIPs, as well as other IgSF CAMs (such as Beat/Sides; Li et al., 2017), can bind multiple partners. Circuit (re)formation in various *Drosophila* neuropils offers an excellent system to overcome redundancy because of the full connectome data, available single cell transcriptomic datasets, and, in the era of CRISPR, genetic ability to perturb the function of multiple genes within a single cell. The zoned structure of the MB is especially intriguing as it can be correlated with layered structures in mammals such as the cerebellum (Li F. et al., 2020).

Due to its awesome genetic power and the wide array of biochemical and imaging techniques, we strongly anticipate breakthroughs in our undertesting of the roles of CAMs in spatiotemporal control of remodeling to arise from *Drosophila*. These are likely to transform our approach to similar mechanisms of neuronal remodeling and (re)wiring in other systems and organisms, in both physiological and pathological contexts.

## Author Contributions

HM and OS wrote the manuscript. Both authors contributed to the article and approved the submitted version.

## Conflict of Interest

The authors declare that the research was conducted in the absence of any commercial or financial relationships that could be construed as a potential conflict of interest.

## Publisher’s Note

All claims expressed in this article are solely those of the authors and do not necessarily represent those of their affiliated organizations, or those of the publisher, the editors and the reviewers. Any product that may be evaluated in this article, or claim that may be made by its manufacturer, is not guaranteed or endorsed by the publisher.

## References

[B1] Alyagor I. Berkun V. Keren-Shaul H. Marmor-Kollet N. David E. Mayseless O. (2018). Combining Developmental and Perturbation-Seq Uncovers Transcriptional Modules Orchestrating Neuronal Remodeling. *Dev. Cell* 47:e36. 10.1016/j.devcel.2018.09.013 30300589 PMC6179959

[B2] Ashley J. Sorrentino V. Lobb-Rabe M. Nagarkar-Jaiswal S. Tan L. Xu S. (2019). Transsynaptic interactions between IgSF proteins DIP-alpha and Dpr10 are required for motor neuron targeting specificity. *Elife* 8:e42690. 10.7554/eLife.42690 30714906 PMC6391064

[B3] Aso Y. Hattori D. Yu Y. Johnston R. M. Iyer N. A. Ngo T. T. (2014). The neuronal architecture of the mushroom body provides a logic for associative learning. *Elife* 3:e04577. 10.7554/eLife.04577 25535793 PMC4273437

[B4] Aso Y. Ray R. P. Long X. Bushey D. Cichewicz K. Ngo T. T. (2019). Nitric oxide acts as a cotransmitter in a subset of dopaminergic neurons to diversify memory dynamics. *Elife* 8:e49257. 10.7554/eLife.49257 31724947 PMC6948953

[B5] Barish S. Nuss S. Strunilin I. Bao S. Mukherjee S. Jones C. D. (2018). Combinations of DIPs and Dprs control organization of olfactory receptor neuron terminals in Drosophila. *PLoS Genet.* 14:e1007560. 10.1371/journal.pgen.1007560 30102700 PMC6107282

[B6] Borgen M. Rowland K. Boerner J. Lloyd B. Khan A. Murphey R. (2017). Axon Termination, Pruning, and Synaptogenesis in the Giant Fiber System of Drosophila melanogaster Is Promoted by Highwire. *Genetics* 205 1229–1245. 10.1534/genetics.116.197343 28100586 PMC5340335

[B7] Bornstein B. Meltzer H. Adler R. Alyagor I. Berkun V. Cummings G. (2021). Transneuronal Dpr12/DIP-delta interactions facilitate compartmentalized dopaminergic innervation of Drosophila mushroom body axons. *EMBO J.* 40:e105763. 10.15252/embj.2020105763 33847376 PMC8204868

[B8] Bornstein B. Zahavi E. E. Gelley S. Zoosman M. Yaniv S. P. Fuchs O. (2015). Developmental Axon Pruning Requires Destabilization of Cell Adhesion by JNK Signaling. *Neuron* 88 926–940. 10.1016/j.neuron.2015.10.023 26586184

[B9] Brennaman L. H. Zhang X. Guan H. Triplett J. W. Brown A. Demyanenko G. P. (2013). Polysialylated NCAM and ephrinA/EphA regulate synaptic development of GABAergic interneurons in prefrontal cortex. *Cereb. Cortex* 23 162–177. 10.1093/cercor/bhr392 22275477 PMC3513957

[B10] Brill M. S. Kleele T. Ruschkies L. Wang M. Marahori N. A. Reuter M. S. (2016). Branch-Specific Microtubule Destabilization Mediates Axon Branch Loss during Neuromuscular Synapse Elimination. *Neuron* 92 845–856. 10.1016/j.neuron.2016.09.049 27773584 PMC5133389

[B11] Carrillo R. A. Ozkan E. Menon K. P. Nagarkar-Jaiswal S. Lee P. T. Jeon M. (2015). Control of Synaptic Connectivity by a Network of Drosophila IgSF Cell Surface Proteins. *Cell* 163 1770–1782. 10.1016/j.cell.2015.11.022 26687361 PMC4720259

[B12] Cavallaro U. Dejana E. (2011). Adhesion molecule signalling: not always a sticky business. *Nat. Rev. Mol. Cell Biol.* 12 189–197. 10.1038/nrm3068 21346732

[B13] Cecchini A. Cornelison D. D. W. (2021). Eph/Ephrin-Based Protein Complexes: the Importance of cis Interactions in Guiding Cellular Processes. *Front. Mol. Biosci.* 8:809364. 10.3389/fmolb.2021.809364 35096972 PMC8793696

[B14] Cheng S. Ashley J. Kurleto J. D. Lobb-Rabe M. Park Y. J. Carrillo R. A. (2019a). Molecular basis of synaptic specificity by immunoglobulin superfamily receptors in Drosophila. *Elife* 8:e41028. 10.7554/eLife.41028 30688651 PMC6374074

[B15] Cheng S. Park Y. Kurleto J. D. Jeon M. Zinn K. Thornton J. W. (2019b). Family of neural wiring receptors in bilaterians defined by phylogenetic, biochemical, and structural evidence. *Proc. Natl. Acad. Sci. U.S.A.* 116 9837–9842. 10.1073/pnas.1818631116 31043568 PMC6525511

[B16] Cocchi E. Drago A. Serretti A. (2016). Hippocampal Pruning as a New Theory of Schizophrenia Etiopathogenesis. *Mol. Neurobiol.* 53 2065–2081. 10.1007/s12035-015-9174-6 25902861

[B17] Cosmanescu F. Katsamba P. S. Sergeeva A. P. Ahlsen G. Patel S. D. Brewer J. J. (2018). Neuron-Subtype-Specific Expression, Interaction Affinities, and Specificity Determinants of DIP/Dpr Cell Recognition Proteins. *Neuron* 100 1385.e6–1400.e6. 10.1016/j.neuron.2018.10.046 30467080 PMC6309224

[B18] Croset V. Treiber C. D. Waddell S. (2018). Cellular diversity in the Drosophila midbrain revealed by single-cell transcriptomics. *Elife* 7:e34550. 10.7554/eLife.34550 29671739 PMC5927767

[B19] del Alamo D. Rouault H. Schweisguth F. (2011). Mechanism and significance of cis-inhibition in Notch signalling. *Curr. Biol.* 21 R40–R47. 10.1016/j.cub.2010.10.034 21215938

[B20] Demyanenko G. P. Mohan V. Zhang X. Brennaman L. H. Dharbal K. E. Tran T. S. (2014). Neural cell adhesion molecule NrCAM regulates Semaphorin 3F-induced dendritic spine remodeling. *J. Neurosci.* 34 11274–11287. 10.1523/JNEUROSCI.1774-14.2014 25143608 PMC4138338

[B21] Dickson B. J. (2002). Molecular mechanisms of axon guidance. *Science* 298 1959–1964. 10.1126/science.1072165 12471249

[B22] Duncan B. W. Murphy K. E. Maness P. F. (2021). Molecular Mechanisms of L1 and NCAM Adhesion Molecules in Synaptic Pruning, Plasticity, and Stabilization. *Front. Cell Dev. Biol.* 9:625340. 10.3389/fcell.2021.625340 33585481 PMC7876315

[B23] Enneking E. M. Kudumala S. R. Moreno E. Stephan R. Boerner J. Godenschwege T. A. (2013). Transsynaptic coordination of synaptic growth, function, and stability by the L1-type CAM Neuroglian. *PLoS Biol.* 11:e1001537. 10.1371/journal.pbio.1001537 23610557 PMC3627646

[B24] Fearnley S. Raja R. Cloutier J. F. (2021). Spatiotemporal expression of IgLON family members in the developing mouse nervous system. *Sci. Rep.* 11:19536. 10.1038/s41598-021-97768-5 34599206 PMC8486791

[B25] Gonda Y. Namba T. Hanashima C. (2020). Beyond Axon Guidance: roles of Slit-Robo Signaling in Neocortical Formation. *Front. Cell Dev. Biol.* 8:607415. 10.3389/fcell.2020.607415 33425915 PMC7785817

[B26] Han C. Jan L. Y. Jan Y. N. (2011). Enhancer-driven membrane markers for analysis of nonautonomous mechanisms reveal neuron-glia interactions in Drosophila. *Proc. Natl. Acad. Sci. U.S.A.* 108 9673–9678. 10.1073/pnas.1106386108 21606367 PMC3111288

[B27] Han C. Wang D. Soba P. Zhu S. Lin X. Jan L. Y. (2012). Integrins regulate repulsion-mediated dendritic patterning of drosophila sensory neurons by restricting dendrites in a 2D space. *Neuron* 73 64–78. 10.1016/j.neuron.2011.10.036 22243747 PMC3269260

[B28] Hong S. Beja-Glasser V. F. Nfonoyim B. M. Frouin A. Li S. Ramakrishnan S. (2016). Complement and microglia mediate early synapse loss in Alzheimer mouse models. *Science* 352 712–716. 10.1126/science.aad8373 27033548 PMC5094372

[B29] Hong W. Luo L. (2014). Genetic control of wiring specificity in the fly olfactory system. *Genetics* 196 17–29. 10.1534/genetics.113.154336 24395823 PMC3872183

[B30] Janssens J. Aibar S. Taskiran I. I. Ismail J. N. Gomez A. E. Aughey G. (2022). Decoding gene regulation in the fly brain. *Nature* 601 630–636. 10.1038/s41586-021-04262-z 34987221

[B31] Jefferis G. S. Marin E. C. Watts R. J. Luo L. (2002). Development of neuronal connectivity in Drosophila antennal lobes and mushroom bodies. *Curr. Opin. Neurobiol.* 12 80–86. 10.1016/s0959-4388(02)00293-3 11861168

[B32] Karis K. Eskla K. L. Kaare M. Taht K. Tuusov J. Visnapuu T. (2018). Altered Expression Profile of IgLON Family of Neural Cell Adhesion Molecules in the Dorsolateral Prefrontal Cortex of Schizophrenic Patients. *Front. Mol. Neurosci.* 11:8. 10.3389/fnmol.2018.00008 29434535 PMC5797424

[B33] Kim M. E. Shrestha B. R. Blazeski R. Mason C. A. Grueber W. B. (2012). Integrins establish dendrite-substrate relationships that promote dendritic self-avoidance and patterning in drosophila sensory neurons. *Neuron* 73 79–91. 10.1016/j.neuron.2011.10.033 22243748 PMC3470655

[B34] Komiyama T. Luo L. (2006). Development of wiring specificity in the olfactory system. *Curr. Opin. Neurobiol.* 16 67–73. 10.1016/j.conb.2005.12.002 16377177

[B35] Kramer R. Rode S. Rumpf S. (2019). Rab11 is required for neurite pruning and developmental membrane protein degradation in Drosophila sensory neurons. *Dev. Biol.* 451 68–78. 10.1016/j.ydbio.2019.03.003 30871987

[B36] Kudumala S. R. Penserga T. Borner J. Slipchuk O. Kakad P. Lee L. H. (2017). Lissencephaly-1 dependent axonal retrograde transport of L1-type CAM Neuroglian in the adult drosophila central nervous system. *PLoS One* 12:e0183605. 10.1371/journal.pone.0183605 28837701 PMC5570280

[B37] Lee K. Doe C. Q. (2021). A locomotor neural circuit persists and functions similarly in larvae and adult Drosophila. *Elife* 10:e69767. 10.7554/eLife.69767 34259633 PMC8298091

[B38] Lee T. Lee A. Luo L. (1999). Development of the Drosophila mushroom bodies: sequential generation of three distinct types of neurons from a neuroblast. *Development* 126 4065–4076. 10.1242/dev.126.18.4065 10457015

[B39] Li F. Lindsey J. W. Marin E. C. Otto N. Dreher M. Dempsey G. (2020). The connectome of the adult Drosophila mushroom body provides insights into function. *Elife* 9:e62576. 10.7554/eLife.62576 33315010 PMC7909955

[B40] Li J. Han S. Li H. Udeshi N. D. Svinkina T. Mani D. R. (2020). Cell-Surface Proteomic Profiling in the Fly Brain Uncovers Wiring Regulators. *Cell* 180 373.e–386.e. 10.1016/j.cell.2019.12.029 31955847 PMC7072036

[B41] Li H. Watson A. Olechwier A. Anaya M. Sorooshyari S. K. Harnett D. P. (2017). Deconstruction of the beaten Path-Sidestep interaction network provides insights into neuromuscular system development. *Elife* 6:e28111. 10.7554/eLife.28111 28829740 PMC5578738

[B42] Li T. Fu T. M. Wong K. K. L. Li H. Xie Q. Luginbuhl D. J. (2021). Cellular bases of olfactory circuit assembly revealed by systematic time-lapse imaging. *Cell* 184 5107.e–5121.e. 10.1016/j.cell.2021.08.030 34551316 PMC8545656

[B43] Luo L. O’Leary D. D. (2005). Axon retraction and degeneration in development and disease. *Annu. Rev. Neurosci.* 28 127–156. 10.1146/annurev.neuro.28.061604.135632 16022592

[B44] Malin J. Desplan C. (2021). Neural specification, targeting, and circuit formation during visual system assembly. *Proc. Natl. Acad. Sci. U.S.A.* 118:e2101823118. 10.1073/pnas.2101823118 34183440 PMC8285955

[B45] Marin E. C. Watts R. J. Tanaka N. K. Ito K. Luo L. (2005). Developmentally programmed remodeling of the Drosophila olfactory circuit. *Development* 132 725–737. 10.1242/dev.01614 15659487

[B46] Matthews B. J. Kim M. E. Flanagan J. J. Hattori D. Clemens J. C. Zipursky S. L. (2007). Dendrite self-avoidance is controlled by Dscam. *Cell* 129 593–604. 10.1016/j.cell.2007.04.013 17482551

[B47] Mayseless O. Berns D. S. Yu X. M. Riemensperger T. Fiala A. Schuldiner O. (2018). Developmental Coordination during Olfactory Circuit Remodeling in Drosophila. *Neuron* 99 1204.e–1215.e. 10.1016/j.neuron.2018.07.050 30146303

[B48] McLaughlin C. N. Brbic M. Xie Q. Li T. Horns F. Kolluru S. S. (2021). Single-cell transcriptomes of developing and adult olfactory receptor neurons in Drosophila. *Elife* 10:e63856. 10.7554/eLife.63856 33555999 PMC7870146

[B49] Meltzer H. Schuldiner O. (2020). With a little help from my friends: how intercellular communication shapes neuronal remodeling. *Curr. Opin. Neurobiol.* 63 23–30. 10.1016/j.conb.2020.01.018 32092689

[B50] Menon K. P. Kulkarni V. Takemura S. Y. Anaya M. Zinn K. (2019). Interactions between Dpr11 and DIP-gamma control selection of amacrine neurons in Drosophila color vision circuits. *Elife* 8:e48935. 10.7554/eLife.48935 31692445 PMC6879306

[B51] Mohan V. Sullivan C. S. Guo J. Wade S. D. Majumder S. Agarwal A. (2019a). Temporal Regulation of Dendritic Spines Through NrCAM-Semaphorin3F Receptor Signaling in Developing Cortical Pyramidal Neurons. *Cereb. Cortex* 29 963–977. 10.1093/cercor/bhy004 29415226 PMC6499012

[B52] Mohan V. Wade S. D. Sullivan C. S. Kasten M. R. Sweetman C. Stewart R. (2019b). Close Homolog of L1 Regulates Dendritic Spine Density in the Mouse Cerebral Cortex Through Semaphorin 3B. *J. Neurosci.* 39 6233–6250. 10.1523/JNEUROSCI.2984-18.2019 31182634 PMC6687901

[B53] Nandagopal N. Santat L. A. Elowitz M. B. (2019). Cis-activation in the Notch signaling pathway. *Elife* 8:e37880. 10.7554/eLife.37880 30628888 PMC6345567

[B54] Ozel M. N. Simon F. Jafari S. Holguera I. Chen Y. C. Benhra N. (2021). Neuronal diversity and convergence in a visual system developmental atlas. *Nature* 589 88–95. 10.1038/s41586-020-2879-3 33149298 PMC7790857

[B55] Ozkan E. Carrillo R. A. Eastman C. L. Weiszmann R. Waghray D. Johnson K. G. (2013). An extracellular interactome of immunoglobulin and LRR proteins reveals receptor-ligand networks. *Cell* 154 228–239. 10.1016/j.cell.2013.06.006 23827685 PMC3756661

[B56] Packard M. Mathew D. Budnik V. (2003). FASt remodeling of synapses in Drosophila. *Curr. Opin. Neurobiol.* 13 527–534. 10.1016/j.conb.2003.09.008 14630214

[B57] Penserga T. Kudumala S. R. Poulos R. Godenschwege T. A. (2019). A Role for Drosophila Amyloid Precursor Protein in Retrograde Trafficking of L1-Type Cell Adhesion Molecule Neuroglian. *Front. Cell Neurosci.* 13:322. 10.3389/fncel.2019.00322 31354437 PMC6640005

[B58] Pollerberg G. E. Thelen K. Theiss M. O. Hochlehnert B. C. (2013). The role of cell adhesion molecules for navigating axons: density matters. *Mech. Dev.* 130 359–372. 10.1016/j.mod.2012.11.002 23183391

[B59] Reichert H. (2009). Evolutionary conservation of mechanisms for neural regionalization, proliferation and interconnection in brain development. *Biol. Lett.* 5 112–116. 10.1098/rsbl.2008.0337 18755655 PMC2657731

[B60] Riccomagno M. M. Kolodkin A. L. (2015). Sculpting neural circuits by axon and dendrite pruning. *Annu. Rev. Cell Dev. Biol.* 31 779–805. 10.1146/annurev-cellbio-100913-013038 26436703 PMC4668927

[B61] Rozbesky D. Verhagen M. G. Karia D. Nagy G. N. Alvarez L. Robinson R. A. (2020). Structural basis of semaphorin-plexin cis interaction. *EMBO J.* 39:e102926. 10.15252/embj.2019102926 32500924 PMC7327498

[B62] Rui M. Bu S. Chew L. Y. Wang Q. Yu F. (2020). The membrane protein Raw regulates dendrite pruning via the secretory pathway. *Development* 147:e37880. 10.1242/dev.191155 32928906

[B63] Rumpf S. Wolterhoff N. Herzmann S. (2019). Functions of Microtubule Disassembly during Neurite Pruning. *Trends Cell Biol.* 29 291–297. 10.1016/j.tcb.2019.01.002 30683460

[B64] Scheffer L. K. Xu C. S. Januszewski M. Lu Z. Takemura S. Y. Hayworth K. J. (2020). A connectome and analysis of the adult Drosophila central brain. *Elife* 9:e57443. 10.7554/eLife.57443 32880371 PMC7546738

[B65] Schuldiner O. Yaron A. (2015). Mechanisms of developmental neurite pruning. *Cell. Mol. Life Sci.* 72 101–119. 10.1007/s00018-014-1729-6 25213356 PMC5086088

[B66] Sekar A. Bialas A. R. de Rivera H. Davis A. Hammond T. R. Kamitaki N. (2016). Schizophrenia risk from complex variation of complement component 4. *Nature* 530 177–183. 10.1038/nature16549 26814963 PMC4752392

[B67] Sergeeva A. P. Katsamba P. S. Cosmanescu F. Brewer J. J. Ahlsen G. Mannepalli S. (2020). DIP/Dpr interactions and the evolutionary design of specificity in protein families. *Nat. Commun.* 11:2125. 10.1038/s41467-020-15981-8 32358559 PMC7195491

[B68] Sullivan C. S. Kumper M. Temple B. S. Maness P. F. (2016). The Neural Cell Adhesion Molecule (NCAM) Promotes Clustering and Activation of EphA3 Receptors in GABAergic Interneurons to Induce Ras Homolog Gene Family, Member A (RhoA)/Rho-associated protein kinase (ROCK)-mediated Growth Cone Collapse. *J. Biol. Chem.* 291 26262–26272. 10.1074/jbc.M116.760017 27803162 PMC5159490

[B69] Tanaka N. K. Tanimoto H. Ito K. (2008). Neuronal assemblies of the Drosophila mushroom body. *J. Comp. Neurol.* 508 711–755. 10.1002/cne.21692 18395827

[B70] Thomas M. S. Davis R. Karmiloff-Smith A. Knowland V. C. Charman T. (2016). The over-pruning hypothesis of autism. *Dev. Sci.* 19 284–305. 10.1111/desc.12303 25845529

[B71] Wang Q. Wang Y. Yu F. (2018). Yif1 associates with Yip1 on Golgi and regulates dendrite pruning in sensory neurons during Drosophila metamorphosis. *Development* 145:dev164475. 10.1242/dev.164475 29769219

[B72] Wang Y. Zhang H. Shi M. Liou Y. C. Lu L. Yu F. (2017). Sec71 functions as a GEF for the small GTPase Arf1 to govern dendrite pruning of Drosophila sensory neurons. *Development* 144 1851–1862. 10.1242/dev.146175 28420712

[B73] Watts R. J. Hoopfer E. D. Luo L. (2003). Axon pruning during Drosophila metamorphosis: evidence for local degeneration and requirement of the ubiquitin-proteasome system. *Neuron* 38 871–885. 10.1016/s0896-6273(03)00295-2 12818174

[B74] Weber T. Stephan R. Moreno E. Pielage J. (2019). The Ankyrin Repeat Domain Controls Presynaptic Localization of Drosophila Ankyrin2 and Is Essential for Synaptic Stability. *Front. Cell Dev. Biol.* 7:148. 10.3389/fcell.2019.00148 31475145 PMC6703079

[B75] Xie Q. Brbic M. Horns F. Kolluru S. S. Jones R. C. Li J. (2021). Temporal evolution of single-cell transcriptomes of Drosophila olfactory projection neurons. *Elife* 10:e63450. 10.7554/eLife.63450 33427646 PMC7870145

[B76] Xu S. Xiao Q. Cosmanescu F. Sergeeva A. P. Yoo J. Lin Y. (2018). Interactions between the Ig-Superfamily Proteins DIP-alpha and Dpr6/10 Regulate Assembly of Neural Circuits. *Neuron* 100 1369.e–1384.e. 10.1016/j.neuron.2018.11.001 30467079 PMC7501880

[B77] Yaniv S. P. Schuldiner O. (2016). A fly’s view of neuronal remodeling. *Wiley Interdiscip. Rev. Dev. Biol.* 5 618–635. 10.1002/wdev.241 27351747 PMC5086085

[B78] Yu F. Schuldiner O. (2014). Axon and dendrite pruning in Drosophila. *Curr. Opin. Neurobiol.* 27 192–198. 10.1016/j.conb.2014.04.005 24793180 PMC5086084

[B79] Zhang H. Wang Y. Wong J. J. Lim K. L. Liou Y. C. Wang H. (2014). Endocytic pathways downregulate the L1-type cell adhesion molecule neuroglian to promote dendrite pruning in Drosophila. *Dev. Cell* 30 463–478. 10.1016/j.devcel.2014.06.014 25158855

[B80] Zong W. Wang Y. Tang Q. Zhang H. Yu F. (2018). Prd1 associates with the clathrin adaptor alpha-Adaptin and the kinesin-3 Imac/Unc-104 to govern dendrite pruning in Drosophila. *PLoS Biol.* 16:e2004506. 10.1371/journal.pbio.2004506 30142146 PMC6126864

